# How Health Habits Influence the Physiological Response During a Physical Activity in Extreme Temperatures?

**DOI:** 10.3390/ijerph17176374

**Published:** 2020-09-01

**Authors:** José Luis Martin-Conty, Francisco Martin-Rodríguez, Juan José Criado-Álvarez, Carlos Alberto Castillo-Sarmiento, Clara Maestre-Miquel, Alicia Mohedano-Moriano, Begoña Polonio-López, Carlos Durantez-Fernández, Miguel Ángel Castro-Villamor, Antonio Viñuela

**Affiliations:** 1 Faculty of Health Sciences, Universidad de Castilla-La Mancha, 45600 Talavera de la Reina, Spain; JoseLuis.MartinConty@uclm.es (J.L.M.-C.); juanjose.criado@uclm.es (J.J.C.-Á.); Alicia.Mohedano@uclm.es (A.M.-M.); Begona.polonio@uclm.es (B.P.-L.); Carlos.durantez@uclm.es (C.D.-F.); Antonio.vinuela@uclm.es (A.V.); 2 Advanced Clinical Simulation Center, School of Medicine, Universidad de Valladolid, 47005 Valladolid, Spain; fmartin@saludcastillayleon.es (F.M.-R.); mcastrovi@saludcastillayleon.es (M.Á.C.-V.); 3 Integrated Care Management of Talavera de la Reina, Health Services of Castilla-La Mancha (SESCAM), 45600 Talavera de la Reina, Spain; 4 Faculty of Physiotherapy, Nursing and Occupational Therapy, Universidad de Castilla-La Mancha, 45071 Toledo, Spain; carlosa.castillo@uclm.es

**Keywords:** eating habits, cardiopulmonary resuscitation, Mediterranean diet, lifestyle, university students

## Abstract

Background: The purpose of the study was to determine to what degree the health habits of university students influence their physiological response during a 10-min high-intensity exercise. Methods: We conducted a cross-sectional cohort study with 59 health science students, in which we analyzed their adherence to a Mediterranean and low-fat diet, as well as their activity levels. We correlated these factors with the physiological response (lactic acid and heart rate) and a series of anthropometric parameters in intense physical activity (cardiopulmonary resuscitation (CPR) for 10 min) in three scenarios: extreme cold, extreme heat and a control situation at room temperature. Results: The results of this study demonstrate that in university students, a greater adherence to the Mediterranean diet was associated with a better response to physical exercise, in this case, 10-min CPR, in hostile environments. Conclusions: Following healthy eating guidelines improves physical performance and delays the appearance of fatigue; both are important aspects for a better performance of CPR.

## 1. Introduction

Obesity and other nutrition-related non-communicable diseases are increasingly becoming major health problems worldwide, particularly in European countries. For example, the global obesity epidemic is linked to an increased risk of some types of cancer, cardiovascular disease and type 2 diabetes mellitus [1].

Health benefits of regular physical activity are highly proven [2], however, studies have reported a continued and progressive decrease in physical activity in young people. In the United States, almost half of university students do not meet the minimum recommendations for physical activity [3]. In other countries, such as Spain, similar patterns are observed with up to 75% of students not meeting the minimum requirements for physical activity [4].

Furthermore, in many cases, students’ daily eating habits are inadequate. A greater consumption of snacks, fast food and a lack of fruits and vegetables together with diminished physical activity are causing increased weight gain with a high prevalence of overweight, obesity and mortality [5,6,7]. This weight gain is not a direct consequence of university attendance, since young people of similar ages in university and non-university environments gain weight equally [8].

The Mediterranean diet was described for the first time by Ancel Keys and is characterized by a high consumption of fruits, vegetables, legumes, unprocessed cereals, olive oil, moderate consumption of dairy products and low consumption of red wine and red meat [9,10,11,12,13]. Its components are rich in monounsaturated fats, complex carbohydrates, fiber and a low consumption of saturated fats [14]. A Mediterranean diet is associated with a healthier lifestyle, a longer life expectancy and a protective effect against certain pathologies such as obesity and cardiovascular diseases. Greater adherence to a Mediterranean diet is also associated with an overall improvement in physical and psychological health, greater longevity and lower levels of obesity and excess weight at all ages [15]. Its benefits for promoting health are amply demonstrated by lower rates of cardiovascular, neoplastic and neurodegenerative diseases and a reduced mortality from these pathologies [16,17]. A higher diet quality and a higher quality of life have been observed in both adolescents and adults who follow a Mediterranean diet [18,19].

Lifestyle and eating habits among the young population are changing in recent years, especially among university students where customs from other countries are being acquired. Factors such as advertising, body worship and different lifestyles are causing many young people to vary their eating patterns, leaving aside a Mediterranean diet [4,20]. In Spain, as in other countries in the Mediterranean region, the diet has moved away from the traditional Mediterranean fare and inclined towards a more industrial diet, with a higher consumption of ultra-processed products and lower intake of fruit and vegetables than recommended in the Mediterranean diet [21,22,23].

It is extensively demonstrated that an active life style and a healthy diet are both related with a better physiological response of the organism (expressed in heart rate and blood lactate) [24,25]. Based on these observations, we decided to analyze whether university students who adhere more to a Mediterranean diet demonstrate a better physiological response to exercise, and to compare whether their habits have a significant impact on different anthropometric parameters. As a result that the training of different health personnel is carried out by our faculty, we decided to use cardiopulmonary resuscitation (CPR) maneuvers as a stress test, since this activity has been found to involve high energy expenditure in young individuals of different physiognomy (19.5 kcal/min in men and 15.1 kcal/min in women) [26] when performed following the requirements of a quality CPR of the American Heart Association (AHA) or the European Resuscitation Council (ERC) (cycles of 30 compressions and two ventilations at a rate of 100–120 chest compressions per minute and a depth of 5–6 cm with the hands placed in the lower center of the sternum) [27,28].

With the intention of better simulating potentially hostile situations with a higher stress and fatigue load for the first responders [29,30], we implemented three simulation scenarios with different temperature and humidity conditions (control, extreme cold and extreme heat).

The main objective of this work was to determine the degree to which the health habits of university students influence their physiological response to a 10-min high-intensity exercise, such as CPR. Our secondary objective was to determine any differences in this physiological response if the exercise was performed in extreme hot or cold conditions. For this, in addition to registering student habits, we analyzed anthropometric parameters and physiological responses of students before and after performing CPR in different conditions. We hypothesize that the students’ diet quality and their levels of physical activity are related to their responses to intense exercise in different conditions.

## 2. Materials and Methods

### 2.1. Study Design

We conducted a cross-sectional cohort study that included students over 18 years of age with basic knowledge of basic life support. All participants were recruited from the degrees of nursing, occupational therapy and speech therapy at the Faculty of Health Sciences of the University of Castilla la Mancha (Talavera de la Reina, Spain). All subjects gave their informed consent for inclusion before they participated in the study. The study was conducted in accordance with the Declaration of Helsinki, and the protocol was approved by the Ethics Committee of the Castilla-La Mancha Health Service (SESCAM) with number 178013/113. The study was carried out at the Faculty of Health Sciences of the University of Castilla la Mancha, in Talavera de la Reina, between May 6 and 10, 2019.

### 2.2. Population

We calculated the sample size accepting an alpha risk of 0.05 and a beta risk of 0.2 in a bilateral contrast. In total, 18 subjects per group (56 subjects) would have been necessary to detect a minimum difference of 0.1 between two groups, assuming that there are three groups and a standard deviation of 0.1 (taken from previous studies) [29,30]. We assumed a loss-to-follow-up rate of 5%, so the final estimated sample was 64 subjects.

A total of 520 nursing, occupational therapy and speech therapy students from the Faculty of Health Sciences of the University of Castilla la Mancha were invited to participate in the study, of whom 220 participants met the requirements to participate in the study.

The exclusion criteria were: not having AHA- or ERC-accredited training for CPR, resting heart rate above 120 beats/minute (bpm) or below 35 bpm; systolic or diastolic blood pressure above 160 or 95 mmHg or systolic blood pressure below 80 mmHg; blood glucose levels below 65 mg/dL; severe visual or hearing impairment (or any type of functional disorder that hinders CPR maneuvers); major surgery in the last 30 days; epilepsy; ongoing treatment for diagnosed infections; electrocardiogram with alterations (arrhythmia or changes in the S-T segment); oxygen saturation below 92%; body mass index above 40 kg/m^2^; body temperature above 38 °C; acute skin diseases; or systemic immune diseases.

**Figure 1:**
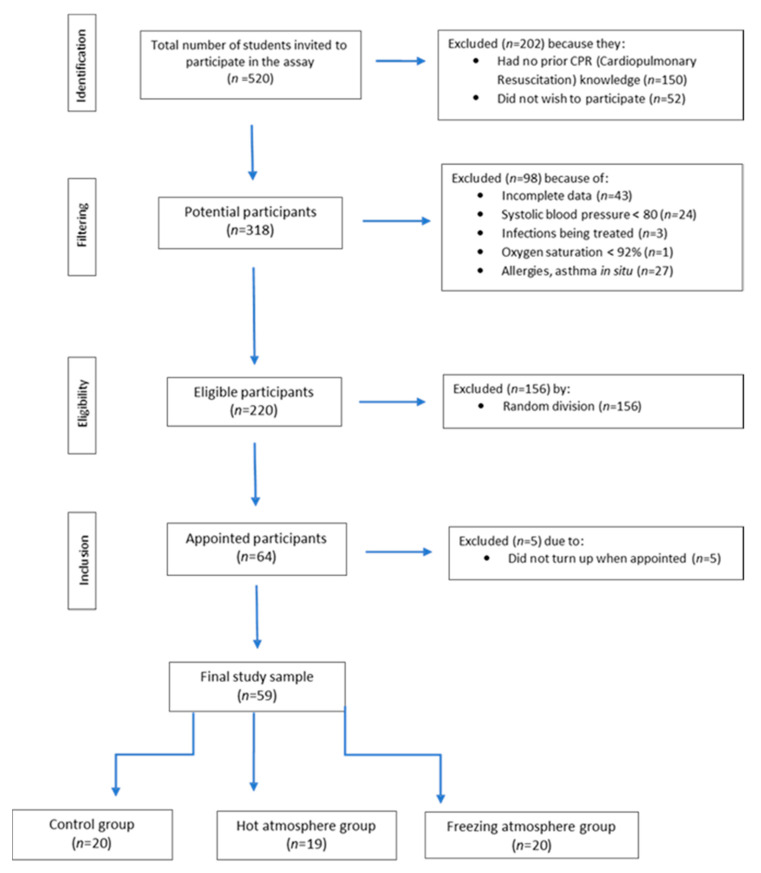
Flow chart of the selection of participants for this study.

As a result that the number of participants meeting the inclusion criteria was very high, a randomization sequence was generated according to the gender stratification created with the XLSTAT^®^ BioMED software for Microsoft Excel^®^ (version 14.4.0; Microsoft Inc.: Redmond, WA, USA). We then called 64 subjects, of whom 59 participants responded to the invitation. These were randomly distributed into three groups for the CPR simulation (20 participants in the control group, 20 participants in the cold environment and 19 participants in the warm environment) (Figure 1).

### 2.3. Study Protocol

Each volunteer included in the study was medically examined. The analysis consisted of directed anamnesis, vital signs, anthropometric study using a bioimpedance scale (Tanita BC-545N; Biologica Tecnologia Medica SL: Barcelona, Spain) (weight, height, BMI, % body fat, muscle mass, bone mass, visceral fat and % body water), spirometry, electrocardiogram and basic analytical determinations (glucose and lactic acid). All these variables were monitored before starting the test and at the end of the study.

To analyze the dietary and lifestyle profiles of the participants, we asked them to complete a series of questionnaires that allowed us to categorize them dichotomously depending on whether their habits were healthy or not. We used the International Physical Activity Questionnaire (IPAQ) [31] to assess the level of activity of the subjects, the KIDMED (Mediterranean Diet Quality Index for children and adolescents) [32] questionnaire to analyze adherence to the Mediterranean diet and the PREDIMED (PREvención con DIeta MEDiterránea) [33] questionnaire for the degree of adherence to a low-fat diet.

For the stress test, we designed three scenarios in which all volunteers had to perform CPR for 10 min without stopping, following the AHA quality standards (30 compressions, two ventilations). Specifically, the simulation scenarios had the following characteristics:

Group 1 (control). Subjects performed basic CPR in a 20-m^2^ laboratory with good lighting, temperature of 21 ℃, and humidity of 60%.

Group 2 (intervention: heat environment). Subjects performed basic CPR in a 20-m^2^ laboratory with good lighting, temperature of 41 °C, and humidity of 98%.

Group 3 (intervention: cold environment). Subjects performed basic CPR in a 20-m^2^ cold storage chamber with good lighting, temperature of −35 °C, and humidity of 80%.

The personal protective equipment used to carry out the stress tests in extreme temperature conditions, both for cold and hot situations, was standardized by the Castilla-La Mancha Emergency Services.

Throughout the CPR simulation, we continuously monitored heart rate, respiratory rate, and electrocardiographic rhythm of each volunteer for study purposes and to immediately detect possible complications. These data were supplied by the Laerdal Resusci Anne^®^ phantom and an X Series^®^ monitor/defibrillator (Zoll: Chelmsford, MA, USA). In addition, every three minutes (minutes 3, 6 and 9 of the test) capillary extractions were performed to carry out a serial curve of lactic acid (AL) using an Accutrend Plus Lactate Meter lactometer (Roche Diagnostics: Mannheim, Germany). These parameters were again measured ten minutes after the end of the simulation.

### 2.4. Data Analysis

Mean ± standard deviation was used to define variables of quantitative and descriptive frequency, whereas categorical variables were described using absolute frequencies with a 95% confidence interval (95% CI).

Statistical analysis was done using the SPSS statistical package, v. 24 (SPSS Inc.: Chicago, IL, USA), establishing a confidence level of 95%.

In the descriptive and inferential statistical analysis, the parameters were used according to the scale of the variable. The quantitative variables were obtained through the bioimpedance scale: weight, muscle mass, body water, bone mass, body fat and visceral fat and BMI (before and after the test). Other quantitative variables related to the subjects’ physiological parameters such as lactate and heart rate were selected for analysis. The qualitative variables corresponded to those that allowed us to categorize the participants according to their dietary habits and lifestyle, that is, adherence to Mediterranean diet, dietary fat intake, IPAQ and BMI (BMI < 25 vs. BMI > 25).

A baseline of the different variables was evaluated at rest to rule out any initial difference between the experimental groups, as well as to confirm that the participants were fit. The first measurement in the experimental protocol was considered the baseline.

We verified the normal distribution of the data using the Kolmogorov–Smirnov test. To make sure that the profile of the subjects in the three groups was the same, we analyzed with the χ^2^ test the ratio of active vs. sedentary subjects (according to IPAQ), subjects with normal weight vs. overweight (according to BMI), subjects who followed the Mediterranean diet vs. subjects who do not follow the Mediterranean diet and subjects with a high fat diet vs. subjects with a low fat diet; adjusting in all cases the female/male ratio.

A t-test was used to study differences in the paired variables. We studied the physiological parameters and the anthropometric parameters by t-test based on the profile of the participants.

The ANOVA statistical test was used to analyze the physiological and anthropometric parameters according to the environmental conditions of the experimental group.

## 3. Results

### 3.1. Descriptive Data

Of the 59 study participants, 27 (45.8%) were male and 32 (54.2%) female, with a total mean age of 21.3 ± 2.7 years. Categorizing them according to their dietary habits and lifestyle, we found that 28 individuals (47.5%) considered themselves sedentary and 31 active (52.5%). Of the sedentary, 12 were men and 16 were women and of the active 15 were men and 16 were women. A total of 35 of them (59.3%) had a BMI < 25, while 24 (40.7%) were overweight. Among the participants with normal weight, 16 were men and 19 women, while those who were overweight 11 were men and 13 were women. Of the 59 participants, 38 (64.4%) admitted to following a Mediterranean diet, while 21 (35.6%) did not follow it. Differentiating between the sexes, we observed that of the 38 that followed it, 20 were men and 18 were women, and among those who did not follow it, the majority were women (14), compared with only seven men. A total of 31 individuals (52.2%) reported following low-fat diets. Among them, 14 were men and 17 women. The rest (47.5%) followed high-fat diets, of whom 13 were men and 15 women (Table 1).

### 3.2. Main Results and Outcome Data

#### 3.2.1. Analysis Based on Adherence to the Mediterranean Diet

Among the participants who followed a Mediterranean diet versus those who did not, we identified no significant differences in heart rate during the test. For lactate levels, the participants who followed a Mediterranean diet showed a decrease from minute 6 (on the verge of significance, *p* = 0.053), which sharpened significantly at minute 9 (*p* = 0.011). The values of both groups were equalized after resting for 10 min (Table 2).

**Figure 2:**
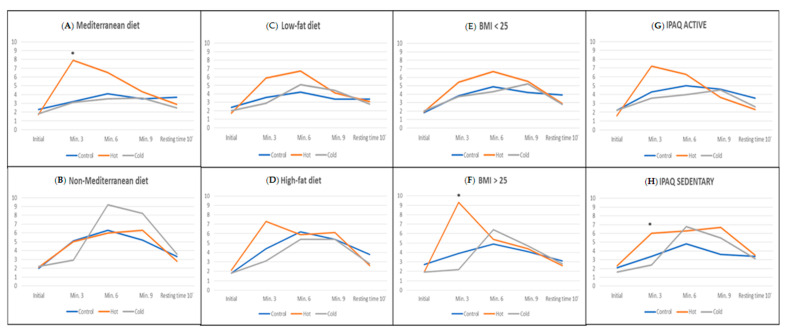
Blood lactate during the test and after 10 min of resting time, considering the environmental conditions and according to the dietary habits and lifestyle of the participants, as follows: (**A**) Mediterranean diet, (**B**) non-Mediterranean diet, (**C**) low-fat diet, (**D**) high-fat diet, (**E**) BMI < 25, (**F**) BMI>25, (**G**) IPAQ active, (**H**) IPAQ sedentary. * Statistically significant differences. Mediterranean diet Min. 3 (*p* = 0.006), BMI > 25 Min. 3 (*p* = 0.008), IPAQ sedentary Min. 3 (*p* = 0.035).

**Figure 3:**
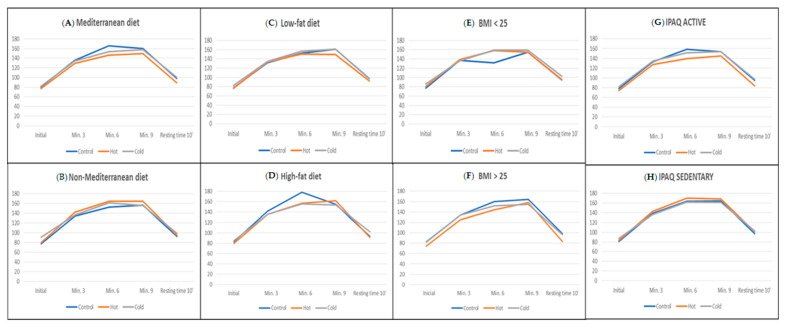
Heart rate during the test and after 10 min of resting time, considering the environmental conditions and according to the dietary habits and lifestyle of the participants, as follows: (**A**) Mediterranean diet, (**B**) non-Mediterranean diet, (**C**) low-fat diet, (**D**) high-fat diet, (**E**) BMI < 25, (**F**) BMI > 25, (**G**) IPAQ active, (**H**) IPAQ sedentary.

Regarding the distribution by environments, we see that the subjects who followed a Mediterranean diet generally presented lower values of lactic acid in the three scenarios, notably in the control group and the cold environment group (Table A1). In Figure 2, we can see how the participants in the warm environment group who followed a Mediterranean diet exhibited much higher lactate levels than the other groups, reaching a significant difference at minute 3 (*p* = 0.006). There were no significant differences in heart rate between the participants in different environmental conditions (Figure 3).

#### 3.2.2. Analysis Based on Adherence to a Low-Fat Diet

No significant differences in heart rate were observed between the participants with adherence to a low-fat diet and those on a high-fat diet. However, there was a significant difference in blood lactate at minute 9 (*p* = 0.051) of those on a low-fat diet. The values were similar after resting for 10 min (Table 2).

Regarding the analysis performed by the different scenarios (Table A2), we see that the results are much better in those participants on a low-fat diet. No significant differences were observed between the scenarios, neither in the individuals on a high-fat diet, nor among those on a low-fat diet (Figure 2 and Figure 3).

#### 3.2.3. Analysis Based on Body Mass Index

The study did not reveal significant associations of the participants’ BMI and their heart rate and blood lactate levels (Table 2). These differences were also not significant in the comparison between participants with normal weight and those with excess weight in the different scenarios (Table A3).

Subjects with BMI > 25 who performed the test in a hot environment presented high levels of lactic acid at minute 3 (9.3 ± 7.2), which was significant compared to participants in other environments (*p* = 0.008). These values were again similar in all scenarios in successive measurements (Figure 2 and Figure 3).

#### 3.2.4. Analysis Based on the Level of Activity According to IPAQ

Of the different variables analyzed, the most significant differences were observed between active and sedentary subjects. Table 2 shows significant differences in heart rate at minute 6 (*p* = 0.019) and at minute 9 (*p* = 0.005) of CPR simulation and even after resting time (more than 10 min) (*p* = 0.03) between active and sedentary participants. No significant differences were observed in lactate levels (Table 2). These results are similar in each of the scenarios, although only the differences found in the hot environment would reach significance (Table A4).

Analyzing the ANOVA by scenarios with active and sedentary individuals, we only found significant differences in the lactic acid of sedentary subjects in the warm environment at minute 3 (*p* = 0.035) compared to the rest of the study groups (Figure 2 and Figure 3).

### 3.3. Other Analyses

In the case of anthropometric measurements, we differentiated between those that are measured on a direct quantitative scale (weight, bone mass, muscle mass), percentages (% body fat, % body water) or indices (BMI, visceral fat index). All of them were measured before starting the test and after finishing. Table 3 shows that there were statistically significant reductions in the mean measurements of weight, body fat, visceral fat and BMI; and a significant increase in muscle mass before as compared to after the simulation.

If we analyze these same parameters taking into account the environmental conditions, we find no statistically significant differences in any of the categories between the individuals who performed the activity in cold conditions. In the control group a decrease in weight, visceral fat and BMI was observed. While all the parameters presented statistically significant changes in the individuals who performed the exercise in hot conditions, these changes correspond to a decrease in the variables of weight, body fat, visceral fat and BMI; and an increase in muscle mass, body water and bone mass.

Table 4 shows the results obtained by analyzing the initial vs. final anthropometric parameters according to the dietary habits and lifestyle of the participants. We found that, independent of whether participants followed a Mediterranean diet, weight and visceral fat between the start and end differed significantly. In the other variables, significant differences were observed in muscle mass, body water, body fat and BMI of individuals who followed a Mediterranean diet, that did not appear in individuals not on a Mediterranean diet.

Individuals on a low-fat diet (31 participants) showed a statistically significant decrease in weight and visceral fat, and a significant increase in body water. Among those on a diet high in fat (28 participants), significant differences were observed in weight, muscle mass, fat and visceral fat and BMI.

In the case of BMI, we found that those participants with a BMI < 25 presented a statistically significant decrease in body fat and visceral fat, and a significant increase in muscle mass. In those with a higher body mass index, in weight, visceral fat and BMI differed significantly.

Finally, we observed that those participants who considered themselves active (31 individuals) showed significant changes in the variables muscle mass, body water, body fat and visceral, whereas among the more sedentary participants, we saw significant differences in weight, visceral fat and BMI.

## 4. Discussion

This study allowed us to discern in university students an association of greater adherence to the Mediterranean diet and an improved ability to do physical exercise (in this case, CPR for 10 min) in hostile environments with high or very low temperatures.

University students do not always follow a healthy diet or healthy eating patterns [34,35,36]. As an example, university students from the USA reported suboptimal eating habits compared to the recommendations [37]. In our sample, 64.4% of the students declared to follow a Mediterranean diet, of whom 52.5% also stated following a low-fat diet. Although many adhere to a Mediterranean diet, the establishment of fast food or ultra-processed food consumption habits cause the levels of saturated fats and sugars to increase. These values are similar to those observed in the student population from other countries in the Mediterranean basin [38].

Tektonidis et al. found that lower adherence to a Mediterranean diet was associated with a higher incidence of heart failure [39], however, other studies found no positive effect of Mediterranean diet on cardiac function [40,41,42]. In our study we did not observe differences in heart rate between participants on a Mediterranean diet and those were not. These data are compatible with previously published literature that is in disagreement regarding the relationship between Mediterranean diet and cardiac function [40,43,44]. Most studies reveal that BMI is the most relevant in relation to heart rate. In our case we did not see a significant relationship, but one reason may be that the exclusion criteria included cardiovascular problems and a BMI > 40, so the participants with a BMI > 25 in our study were healthy. Studies similar to ours with participants with a BMI > 25 performing physical exercise for a more extended time would be necessary to identify this association.

Significant differences were observed in lactate levels. We observed, from minute 6 onwards, clear differences between participants who followed a Mediterranean diet and those who did not. These differences were significant at minute 9. Lactate decreased much earlier and with values similar to those when already at rest. In the analysis by environments, we observe that the lactate value was significant at minute 3 in a hot environment, unlike in a cold environment or at room temperature. Lactic acid increases during physical exercise when energy for the muscle is generated [45]. Similarly, participants with a BMI value >25 in a hot environment have significant values that are not observed in other groups of participants. These data are compatible with studies showing how a higher BMI slows the release of glucose in the blood and consequently increases the levels of lactic acid in the blood [46]. These increased values were also observed in the cold at minute 6 but were not statistically significant.

In contrast, we observed significant differences between more active participants and more sedentary ones from minute 6 onwards and even in the recovery from physical effort. The behavior of lactate levels in sedentary participants in a hot environment was the same as in those with BMI > 25. This increase in minute 3 can be also explained by the lesser adequacy of their muscles for physical work. The data were comparable in the cold environment, in which the lactate values were also higher, but the participants reached these values as of minute 6, although they were not significant as in the case of BMI.

Lifestyle and eating habits are directly associated with physical and mental health [47,48,49,50,51,52,53]. Due to the importance they have in the population, first responders are an important study population for assessing nutritional intervention and optimal physical activity in time and quality.

Students who adhere to a Mediterranean diet consider their health to be in better condition, have improved sleep and report low levels of stress [47]. These observations are supported by our results, as the participants in our study showed greater resistance to physical exercise than those who did not follow a Mediterranean diet, both in standard conditions and in more hostile environments.

The fact that the participants’ eating and exercise habits were self-reported in different questionnaires may lead to participants reporting healthier habits than they actually follow, posing a potential source of bias. Regarding the study design, we chose a CPR simulation as a high-intensity exercise given its novel nature, but various confounding factors might have influenced the participants’ performance, such as the continuous observation by the researchers during the CPR, the continuous monitoring of constants on the simulator monitor or the momentary interruption of the test for lactate monitoring during minutes 3, 6 and 9 of CPR, which was performed during the ventilation phase to minimize its impact. Likewise, a participant selection bias is possible, since only students who were interested in the topic volunteered.

Limitations: The size of the sample could be bigger and varied; that could guarantee a better representativeness of the outcomes. There are also different confounding factors that could distract the participant during the experiment (constant watchfulness of researchers, monitoring physiological parameters and others). In addition, using questionnaires for requesting habits could cause bias on the study if the participants do not answer honestly.

Strengths: a very new aspect provided in this study is the classification of the groups by climate conditions on the experiment developed, as well as using CPR as a physical activity to evaluate metabolic fatigue according to the life habits of the participants. This forces us to make recommendations about healthy life styles to the rescuers who could intervene on a cardiopulmonary resuscitation.

## 5. Conclusions

Establishing regulated courses on nutrition among first responders would be advisable in view of the results of this study. We saw a better response to fatigue in those who followed a Mediterranean and low-fat diet. We suggest employing policies and campaigns targeting first responders to improve their eating habits and thus increase their stamina during CPR.

## Figures and Tables

**Table 1:** Description of the study population.

Variables	Control	Hot	Cold	Total		*p*-Value	
	*n* (%)	*n* (%)	*n* (%)	*n*			
IPAQ	Sedentary		10	9	9	28	0.951
			35.7%	32.1%	32.1%		
	Active		10	10	11	31	
			32.3%	32.3%	35.5%		
BMI	Normal weight		11	13	11	35	0.618
			31.4%	37.1%	31.4%		
	Excess weight		9	6	9	24	
			37.5%	25%	37.5%		
Mediterranean diet	No		7	8	6	21	0.731
			33.3%	38.1%	28.6%		
	Yes		13	11	14	38	
			34.2%	28.9%	36.8%		
Fat diet	High		7	10	11	28	0.386
			25%	35.7%	39.3%		
	Low		13	9	9	31	
			41.9%	29%	29%		

**Table 2:** Physiological parameters (heart rate/blood lactate) according to the dietary habits and lifestyle of the participants (Mediterranean diet, adherence to a fat diet, BMI, International Physical Activity Questionnaire (IPAQ)).

	Mediterranean Diet			Adherence to a Fat Diet			BMI			IPAQ		
	Yes (*n* = 38)	No (*n* = 21)		Low (*n* = 31)	High (*n* = 28)		<25 (*n* = 35)	>25 (*n* = 24)		Active (*n* = 31)	Sedentary (*n* = 28)	
	Mean ± SD	Mean ± SD	*p*-Value	Mean ± SD	Mean ± SD	*p*-Value	Mean ± SD	Mean ± SD	*p*-Value	Mean ± SD	Mean ± SD	*p*-Value
Heart Rate												
Initial	79.9 ± 15.0	82.0 ± 13.8	0.599	78.7 ± 13.1	82.8 ± 15.8	0.289	80.9 ± 13.8	80.2 ± 15.7	0.862	78.0 ± 13.8	83.6 ± 14.9	0.135
Min. 3	133.6 ± 18.0	138.1 ± 10.9	0.305	133.2 ± 18.0	137.4 ± 13.2	0.322	137.4 ± 16.5	131.9 ± 14.8	0.194	131.7 ± 17.0	139.1 ± 13.8	0.074
Min. 6	155.4 ± 26.5	159.9 ± 23.5	0.519	152.6 ± 25.3	161.9 ± 25.1	0.164	159.7 ± 25.6	153.2 ± 25.1	0.340	149.7 ± 26.7	165.1 ± 21.5	0.019 *
Min. 9	156.2 ± 21.3	159.1 ± 16.8	0.584	157.5 ± 20.2	156.9 ± 19.6	0.918	156.1 ± 21.0	158.9 ± 17.9	0.595	150.6 ± 21.8	164.5 ± 14.2	0.005 *
Resting time 10 min.	96.3 ± 16.2	95.0 ± 12.1	0.738	95.8 ± 13.5	95.9 ± 16.4	0.982	97.3 ± 13.4	93.7 ± 16.6	0.375	91.9 ± 15.9	100.2 ± 12.3	0.030 *
Blood Lactate												
Initial	1.9 ± 1.3	2.1 ± 1.2	0.643	2.1 ± 1.4	1.9 ± 1.1	0.672	1.9 ± 1.0	2.2 ± 1.5	0.385	2.0 ± 1.4	2.0 ± 1.1	0.940
Min. 3	4.5 ± 4.2	4.4 ± 3.0	0.946	4.1 ± 3.3	4.9 ± 4.3	0.386	4.4 ± 3.2	4.6 ± 4.6	0.825	5.0 ± 4.3	3.9 ± 3.2	0.287
Min. 6	4.6 ± 3.3	7.0 ± 4.9	0.053 *	5.2 ± 3.5	5.8 ± 4.6	0.561	5.4 ± 3.4	5.6 ± 4.9	0.881	5.0 ± 3.5	5.9 ± 4.6	0.418
Min. 9	3.8 ± 2.6	6.5 ± 4.1	0.011 *	3.9 ± 2.7	5.6 ± 3.8	0.051 *	5.0 ± 3.6	4.4 ± 3.1	0.525	4.3 ± 2.6	5.2 ± 4.1	0.300
Resting time 10 min.	3.0 ± 1.7	3.2 ± 1.6	0.682	3.1 ± 1.7	3.0 ± 1.6	0.743	3.2 ± 1.7	2.9 ± 1.7	0.538	2.8 ± 1.8	3.3 ± 1.5	0.243

* Statistically significant differences. SD, standard deviation.

**Table 3:** Anthropometric measurements (weight, bone mass, muscle mass, body water, body fat, visceral fat, BMI) before starting and after finishing the test, considering the environmental conditions.

Variables	Total (*n* = 59)		Control (*n* = 20)		Hot (*n* = 19)		Cold (*n* = 20)	
	Mean ± SD	*p*-Value	Mean ± SD	*p*-Value	Mean ± SD	*p*-Value	Mean ± SD	*p*-Value
Initial weight (kg)	71.59 ± 15.58	0.000 *	72.76 ± 14.65	0.007 *	67.71 ± 10.93	0.022 *	74.10 ± 19.74	0.268
Final weight (kg)	71.36 ± 15.41		72.49 ± 14.51		67.44 ± 10.71		73.97 ± 19.54	
Initial muscle mass (kg)	47.21 ± 6.17	0.018 *	46.47 ± 5.95	0.500	46.44 ± 4.90	0.001 *	48.68 ± 7.40	0.527
Final muscle mass (kg)	47.90 ± 5.90		46.90 ± 6.22		47.83 ± 4.38		48.96 ± 6.88	
Initial body water (%)	51.99 ± 8.53	0.087	49.20 ± 11.82	0.288	54.21 ± 4.86	0.000 *	52.67 ± 6.82	0.996
Final body water (%)	53.36 ± 6.32		51.46 ± 5.38		56.10 ± 4.95		52.66 ± 7.62	
Initial bone mass (kg)	2.54 ± 0.31	0.203	2.50 ± 0.29	0.725	2.48 ± 0.24	0.006 *	2.62 ± 0.37	0.781
Final bone mass (kg)	2.56 ± 0.29		2.51 ± 0.29		2.55 ± 0.22		2.61 ± 0.35	
Initial body fat (%)	29.07 ± 8.17	0.000 *	31.48 ± 7.49	0.054	26.62 ± 6.92	0.001 *	29.00 ± 9.50	0.092
Final body fat (%)	27.56 ± 8.68		30.13 ± 8.16		24.39 ± 7.01		28.00 ± 9.98	
Initial visceral fat	3.74 ± 2.90	0.000 *	4.30 ± 2.79	0.019 *	2.71 ± 1.79	0.001 *	4.15 ± 3.65	0.101
Final visceral fat	3.25 ± 2.53		3.68 ± 2.33		2.32 ± 1.78		3.73 ± 3.13	
Initial BMI	24.76 ± 4.44	0.001 *	25.22 ± 4.67	0.007 *	23.64 ± 2.90	0.027 *	25.37 ± 5.35	0.433
Final BMI	24.68 ± 4.38		25.12 ± 4.59		23.55 ± 2.83		25.34 ± 5.29	

* Statistically significant differences. SD, standard deviation.

**Table 4:** Anthropometric measurements (weight, bone mass, muscle mass, body water, body fat, visceral fat, BMI) before starting and after finishing the test, according to the dietary habits and lifestyle of the participants (Mediterranean diet, adherence to a fat diet, BMI, IPAQ).

Variables	Mediterranean Diet		Adherence to a Fat Diet		BMI		IPAQ	
	Yes (*n* = 38)	No (*n* = 21)	Low (*n* = 31)	High (*n* = 28)	<25 (*n* = 35)	>25 (*n* = 24)	Active (*n* = 31)	Sedentary (*n* = 28)
	Mean ± SD	Mean ± SD	Mean ± SD	Mean ± SD	Mean ± SD	Mean ± SD	Mean ± SD	Mean ± SD
Initial weight (kg)	74.76 ± 16.19	65.85 ± 12.83	71.55 ± 11.70	71.63 ± 19.21	62.62 ± 8.04	84.67 ± 14.68	68.84 ± 13.98	74.64 ± 16.94
Final weight (kg)	74.50 ± 16.02	65.69 ± 12.73	71.38 ± 11.66	71.34 ± 18.95	62.56 ± 8.05	84.20 ± 14.60	68.70 ± 13.92	74.31 ± 16.67
*p*-value	0.004 *	0.041 *	0.041 *	0.005 *	0.336	0.000 *	0.132	0.000 *
Initial muscle mass (kg)	48.19 ± 6.30	45.44 ± 5.67	47.45 ± 5.59	46.94 ± 6.86	44.65 ± 4.53	50.94 ± 6.43	47.10 ± 6.14	47.33 ± 6.32
Final muscle mass (kg)	49.07 ± 6.22	45.78 ± 4.71	47.86 ± 4.76	47.93 ± 7.05	45.77 ± 4.53	51.00 ± 6.37	48.00 ± 5.78	47.78 ± 6.14
*p*-value	0.002 *	0.600	0.375	0.003 *	0.002 *	0.895	0.002 *	0.390
Initial body water (%)	50.62 ± 9.64	54.46 ± 5.40	52.56 ± 5.40	51.35 ± 11.10	56.09 ± 4.21	46.01 ± 9.73	54.31 ± 5.94	49.42 ± 10.21
Final body water (%)	52.83 ± 6.04	54.32 ± 6.84	53.23 ± 5.94	53.51 ± 6.83	56.87 ± 4.83	48.24 ± 4.51	55.44 ± 5.70	51.06 ± 6.27
*p*-value	0.043 *	0.903	0.041 *	0.196	0.231	0.204	0.002 *	0.324
Initial bone mass (kg)	2.59 ± 0.31	2.44 ± 0.29	2.54 ± 0.26	2.53 ± 0.36	2.42 ± 0.23	2.71 ± 0.34	2.54 ± 0.30	2.54 ± 0.32
Final bone mass (kg)	2.62 ± 0.30	2.45 ± 0.24	2.55 ± 0.24	2.56 ± 0.34	2.45 ± 0.22	2.71 ± 0.32	2.56 ± 0.29	2.56 ± 0.29
*p*-value	0.133	0.706	0.587	0.213	0.123	0.883	0.405	0.345
Initial body fat (%)	30.57 ± 8.36	26.36 ± 7.24	29.26 ± 7.42	28.86 ± 9.07	24.38 ± 5.57	35.91 ± 6.34	27.08 ± 8.03	31.28 ± 7.89
Final body fat (%)	28.87 ± 8.56	25.20 ± 8.58	28.19 ± 8.35	26.87 ± 9.13	22.10 ± 5.12	35.53 ± 6.30	25.04 ± 7.93	30.35 ± 8.75
*p*-value	0.000 *	0.205	0.056	0.000 *	0.000 *	0.516	0.000 *	0.107
Initial visceral fat	3.99 ± 2.78	3.29 ± 3.12	3.84 ± 2.73	3.63 ± 3.13	2.00 ± 1.13	6.27 ± 2.85	2.94 ± 2.22	4.63 ± 3.33
Final visceral fat	3.66 ± 2.69	2.52 ± 2.08	3.24 ± 2.03	3.27 ± 3.02	1.73 ± 1.00	5.48 ± 2.44	2.68 ± 2.12	3.89 ± 2.82
*p*-value	0.000 *	0.028 *	0.011 *	0.000 *	0.000 *	0.008 *	0.000 *	0.005 *
Initial BMI	25.59 ± 4.75	23.27 ± 3.46	24.56 ± 3.40	24.99 ± 5.43	21.96 ± 1.71	28.85 ± 4.01	23.69 ± 3.52	25.95 ± 5.09
Final BMI	25.49 ± 4.66	23.22 ± 3.45	24.50 ± 3.37	24.89 ± 5.33	21.94 ± 1.72	28.69 ± 3.97	23.65 ± 3.50	25.84 ± 4.99
*p*-value	0.006 *	0.076	0.059	0.008 *	0.400	0.000 *	0.169	0.001 *

* Statistically significant differences. SD, standard deviation.

## Appendix A

**Table A1:** Physiological parameters (heart rate and blood lactate) based on the adherence to a Mediterranean diet, considering the environmental conditions.

Variables	Mediterranean Diet								
	Control (*n* = 20)			Hot (*n* = 19)			Cold (*n* = 20)		
	Yes (*n* = 7)	No (*n* = 13)		Yes (*n* = 8)	No (*n* = 11)		Yes (*n* = 6)	No (*n* = 14)	
	Mean ± SD	Mean ± SD	*p*-Value	Mean ± SD	Mean ± SD	*p*-Value	Mean ± SD	Mean ± SD	*p*-Value
Heart Rate									
Initial	80.5 ± 17.4	77.4 ± 11.7	0.679	77.2 ± 14.0	79.7 ± 12.4	0.696	81.5 ± 14.1	90.5 ± 16.1	0.227
Min. 3	136.2 ± 17.6	134.5 ± 9.9	0.817	129.4 ± 19.4	142.1 ± 10.7	0.116	134.4 ± 18.1	136.9 ± 12.3	0.764
Min. 6	165.5 ± 25.2	152.9 ± 28.6	0.323	146.1 ± 29.4	164.7 ± 24.5	0.165	153.4 ± 23.9	161.7 ± 17.1	0.451
Min. 9	160.3 ± 17.9	156.2 ± 15.8	0.619	149.8 ± 23.2	164.3 ± 17.8	0.159	157.3 ± 23.0	155.6 ± 17.7	0.877
Resting time 10 min.	98.2 ± 12.6	92.1 ± 11.2	0.302	89.3 ± 18.2	94.7 ± 10.2	0.465	100.1 ± 16.9	98.6 ± 16.2	0.859
Blood Lactate									
Initial	2.3 ± 1.7	2.0 ± 1.1	0.708	1.7 ± 0.7	2.1 ± 1.4	0.505	1.8 ± 1.1	2.2 ± 1.4	0.473
Min. 3	3.2 ± 1.7	5.1 ± 2.0	0.041 *	7.9 ± 6.1	5.0 ± 4.0	0.206	3.1 ± 2.6	2.9 ± 2.0	0.883
Min. 6	4.1 ± 2.5	6.3 ± 3.1	0.114	6.5 ± 4.5	6.0 ± 4.1	0.811	3.5 ± 2.3	9.2 ± 7.2	0.116
Min. 9	3.5 ± 2.7	5.2 ± 2.3	0.206	4.3 ± 1.6	6.3 ± 4.6	0.266	3.6 ± 3.1	8.2 ± 4.9	0.021 *
Resting time 10 min.	3.7 ± 1.5	3.3 ± 0.9	0.578	2.9 ± 1.5	2.8 ± 1.6	0.940	2.5 ± 2.0	3.6 ± 2.3	0.301

* Statistically significant differences. SD, standard deviation.

**Table A2:** Physiological parameters (heart rate and blood lactate) based on the adherence to a fat-diet, considering the environmental conditions.

Variables	Adherence to a Fat-Diet								
	Control (*n* = 20)			Hot (*n* = 19)			Cold (*n* = 20)		
	Low (*n* = 13)	High (*n* = 7)		Low (*n* = 9)	High (*n* = 10)		Low (*n* = 9)	High (*n* = 11)	
	Mean ± SD	Mean ± SD	*p*-Value	Mean ± SD	Mean ± SD	*p*-Value	Mean ± SD	Mean ± SD	*p*-Value
Heart Rate									
Initial	77.3 ± 13.4	83.2 ± 19.2	0.430	76.6 ± 9.0	79.8 ± 16.2	0.607	82.8 ± 16.3	85.2 ± 14.4	0.733
Min. 3	132.1 ± 17.6	142.1 ± 4.9	0.161	133.7 ± 18.6	135.8 ± 16.7	0.799	134.4 ± 20.0	135.8 ± 13.6	0.858
Min. 6	151.7 ± 26.0	178.6 ± 17.5	0.026 *	150.3 ± 27.6	157.2 ± 30.1	0.613	156.3 ± 24.4	155.6 ± 21.0	0.947
Min. 9	161.1 ± 17.2	154.9 ± 16.9	0.448	149.1 ± 19.6	162.1 ± 22.9	0.207	160.7 ± 24.3	153.6 ± 18.7	0.474
Resting time 10 min.	97.6 ± 10.9	93.2 ± 14.9	0.466	92.9 ± 12.1	90.9 ± 18.3	0.833	96.6 ± 18.4	102.1 ± 14.7	0.467
Blood Lactate									
Initial	2.4 ± 1.8	1.8 ± 1.0	0.470	1.7 ± 0.8	2.1 ± 1.2	0.412	2.0 ± 1.2	1.8 ± 1.1	0.742
Min. 3	3.6 ± 2.0	4.4 ± 2.1	0.432	5.9 ± 4.9	7.3 ± 5.9	0.580	2.9 ± 2.3	3.1 ± 2.6	0.852
Min. 6	4.2 ± 2.7	6.2 ± 2.6	0.126	6.7 ± 3.7	5.9 ± 4.8	0.702	5.1 ± 4.1	5.4 ± 5.7	0.892
Min. 9	3.4 ± 2.6	5.4 ± 2.4	0.111	4.1 ± 1.8	6.1 ± 4.0	0.202	4.4 ± 3.7	5.4 ± 4.6	0.620
Resting time 10 min.	3.4 ± 1.6	3.8 ± 0.7	0.489	3.1 ± 1.5	2.6 ± 1.5	0.498	2.8 ± 2.2	2.8 ± 2.1	0.976

* Statistically significant differences. SD, standard deviation.

**Table A3:** Physiological parameters (heart rate and blood lactate) based on BMI, considering the environmental conditions.

Variables	BMI								
	Control (*n* = 20)			Hot (*n* = 19)			Cold (*n* = 20)		
	<25 (*n* = 11)	>25 (*n* = 9)		<25 (*n* = 13)	>25 (*n* = 6)		<25 (*n* = 11)	>25 (*n* = 9)	
	Mean ± SD	Mean ± SD	*p*-Value	Mean ± SD	Mean ± SD	*p*-Value	Mean ± SD	Mean ± SD	*p*-Value
Heart Rate									
Initial	76.7 ± 14.5	82.7 ± 16.7	0.398	80.2 ±12.5	74.1 ± 14.4	0.362	86.0 ± 14.3	81.8 ± 16.2	0.547
Min. 3	136.8 ± 19.0	134.1 ± 9.1	0.710	139.2 ± 16.2	125.2 ± 16.5	0.102	136.0 ± 15.4	134.1 ± 18.2	0.803
Min. 6	161.8 ± 27.7	160.2 ± 26.3	0.894	158.4 ± 28.4	144.2 ± 28.1	0.325	159.0 ± 22.0	152.1 ± 22.6	0.504
Min. 9	154.7 ± 20.1	164.1 ± 10.9	0.224	154.9 ± 21.5	158.1 ± 24.6	0.781	158.9 ± 23.1	154.3 ± 19.3	0.640
Resting time 10 min.	94.5 ± 11.8	98.0 ± 13.1	0.545	95.3 ± 11.2	83.5 ± 20.5	0.118	102.3 ± 16.8	96.4 ± 15.9	0.434
Blood Lactate									
Initial	1.8 ± 0.7	2.7 ± 2.1	0.289	1.9 ± 1.0	1.9 ± 1.3	0.983	2.0 ± 1.4	1.9 ± 0.9	0.922
Min. 3	3.8 ± 1.9	3.9 ± 2.3	0.942	5.4 ± 4.1	9.3 ± 7.2	0.273	3.7 ± 2.9	2.2 ± 1.2	0.165
Min. 6	4.9 ± 2.9	4.9 ± 2.9	0.984	6.7 ± 4.0	5.4 ± 4.8	0.524	4.3 ± 2.7	6.4 ± 6.8	0.422
Min. 9	4.2 ± 2.6	4.1 ± 2.9	0.944	5.5 ± 3.9	4.4 ± 1.1	0.545	5.2 ± 4.2	4.7 ± 4.3	0.787
Resting time 10 min.	3.9 ± 1.6	3.1 ± 0.9	0.256	2.9 ± 1.4	2.6 ± 1.8	0.695	2.8 ± 2.0	2.8 ± 2.3	0.959

* Statistically significant differences. SD, standard deviation.

**Table A4:** Physiological parameters (heart rate and blood lactate) based on the physical activity level, considering the environmental conditions.

	IPAQ								
	Control (*n* = 20)			Hot (*n* = 19)			Cold (*n* = 20)		
	Active (*n* = 10)	Sedentary (*n* = 10)		Active (*n* = 9)	Sedentary (*n* = 10)		Active (*n* = 9)	Sedentary (*n* = 11)	
	Mean ± SD	Mean ± SD	*p*-Value	Mean ± SD	Mean ± SD	*p*-Value	Mean ± SD	Mean ± SD	*p*-Value
Heart rate									
Initial	77.7 ± 15.4	81.2 ± 16.0	0.625	74.0 ± 12.7	83.1 ± 12.4	0.133	81.9 ± 13.5	87.0 ± 16.8	0.464
Min. 3	132.7 ± 16.5	138.5 ± 13.7	0.401	127.6 ± 19.6	142.7 ± 9.8	0.052 *	134.4 ± 15.9	136.0 ± 17.7	0.835
Min. 6	158.5 ± 28.2	163.7 ± 25.8	0.668	139.6 ± 26.0	169.8 ± 22.6	0.016 *	150.9 ± 25.3	162.0 ± 16.5	0.272
Min. 9	153.5 ± 20.8	164.3 ± 10.3	0.167	144.9 ± 19.4	168.2 ± 18.1	0.016 *	153.2 ± 25.5	161.1 ± 14.4	0.419
Resting time 10 min.	94.9 ± 12.9	97.3 ± 12.1	0.674	83.2 ± 14.3	101.0 ± 10.3	0.007 *	97.1 ± 17.7	102.7 ± 14.8	0.460
Blood Lactate									
Initial	2.2 ± 1.8	2.1 ± 1.3	0.913	1.6 ± 0.6	2.3 ± 1.3	0.196	2.2 ± 1.4	1.6 ± 0.7	0.328
Min. 3	4.3 ± 1.9	3.4 ± 2.0	0.332	7.2 ± 6.4	6.0 ± 4.2	0.655	3.6 ± 2.8	2.4 ± 1.8	0.269
Min. 6	5.0 ± 3.0	4.8 ± 2.8	0.845	6.3 ± 4.8	6.3 ± 3.7	0.977	4.0 ± 2.4	6.8 ± 6.7	0.261
Min. 9	4.6 ± 2.4	3.6 ± 2.9	0.449	3.7 ± 1.5	6.7 ± 4.1	0.049 *	4.5 ± 3.6	5.5 ± 4.9	0.612
Resting time 10 min.	3.6 ± 1.8	3.4 ± 0.7	0.767	2.3 ± 1.2	3.5 ± 1.6	0.097	2.6 ± 2.0	3.1 ± 2.2	0.573

* Statistically significant differences. SD, standard deviation.
